# The role of the MicroBiome in PANCreatic cancer and its precursors— the study protocol of the MiBiPanc systematic review and meta-analysis

**DOI:** 10.1186/s13643-025-02910-3

**Published:** 2025-07-18

**Authors:** My-Lan Pianka, Alexander Werba, Samuel Zimmermann, Johannes A. Vey, Eva Kalkum, Solveig Tenckoff, Andrew Tony-Odigie, Christoph W. Michalski, Frank Pianka

**Affiliations:** 1 Department of Infectious Diseases, Medical Microbiology and Hygiene, Medical Faculty Heidelberg, Heidelberg University, Heidelberg, Germany; 2 Study Center of the German Society of Surgery, University of Heidelberg, Heidelberg, Germany; 3 Institute of Medical Biometry, University of Heidelberg, Heidelberg, Germany; 4 Department of General, Visceral and Transplantation Surgery, University of Heidelberg, Heidelberg, Germany

**Keywords:** Pancreatic cancer, Microbiome, Gut microbiome, Systematic review

## Abstract

**Background:**

Pancreatic cancer is the third leading cause of cancer-related death in Northern America and fourth in Europe. Emerging evidence suggests that the pancreatic microbiome may play a significant role in the development and progression of this disease. Although the human microbiota contributes to health by supporting nutritional and hormonal homeostasis, modulating inflammation, detoxifying harmful compounds, and producing beneficial metabolites, several studies have implicated its crucial modulatory role in numerous diseases, including cancer. The main objective of this review is to investigate the specific relationship between the microbiome and pancreatic carcinogenesis.

**Methods:**

A comprehensive literature search will identify studies examining the microbiome in human samples of saliva, pancreatic fluid, bile, pancreatic tissue, and feces of patients with chronic pancreatitis, precancerous pancreatic lesions, and pancreatic cancer. Studies differentiating bacteria to at least the genus level will be prioritized. Eligible studies include randomized controlled trials and observational studies analyzing the human microbiome in patients with chronic pancreatitis, pancreatic precursor lesions, or pancreatic cancer compared to healthy controls. Studies analyzing nonhuman samples, single bacterial strains, or lacking comparator groups will be excluded. The following databases will be searched without any restrictions to the publication date up until December 2024: the Cochrane Central Register of Controlled Trials (CENTRAL), MEDLINE (via PubMed), Embase, and Web of Science. Animal studies, case reports, and studies not reporting analyses of human samples are excluded. Details regarding blinding, risk of bias, and funding sources will be extracted and assessed. The main outcomes include the bacterial diversity in each sample type (stool, saliva, bile, intratumoral, and tissue) itemized for each diagnosis, identifying differentially abundant or depleted taxa, and evaluating the correlation of specific bacteria with disease prevention or progression and clinical outcomes. Data extraction will be performed independently by two reviewers. Risk-of-bias assessment will be performed using Cochrane tools appropriate for each study design. Comparisons will be analyzed by descriptive statistics, and meta-analyses will be performed when applicable. The review will be conducted according to the Preferred Reporting Items for Systematic reviews and Meta-Analyses (PRISMA) guidelines.

**Discussion:**

In summary, this systematic review aims to synthesize studies analyzing microbiome profiles in patients with chronic pancreatitis, precursor lesions, and pancreatic cancer, focusing on identifying bacterial diversity and specific taxa related to disease progression and development of cancer in comparison to healthy controls and will include a thorough critical appraisal of the available literature. Anticipated limitations include heterogeneity in microbiome sampling methods and potential variability in taxonomic resolution across studies.

**Systematic review registration:**

PROSPERO CRD42023487995.

**Supplementary Information:**

The online version contains supplementary material available at 10.1186/s13643-025-02910-3.

## Introduction

Pancreatic cancer ranks as the sixth most common cause of cancer-related mortality worldwide and third in Northern America [1]. The most prevalent type is pancreatic ductal adenocarcinoma (PDAC). Its 5-year survival rate has remained unchanged at approximately 10% throughout the past few decades, which is mainly due to late diagnosis and the limited therapeutic efficacy of current treatments [2]. Recent therapeutic strategies involve a multidisciplinary approach, with surgery remaining the only potentially curative option. This enhances the urgent need for novel preventive and early diagnostic measures.

The pathogenesis of pancreatic cancer is multifaceted and influenced by a variety of individual and environmental risk factors, including genetic predisposition, diet, tobacco and alcohol consumption, obesity, and diabetes mellitus. Chronic pancreatitis (CP) may also increase the risk of developing pancreatic cancer, as metaplasia of pancreatic acinar cells has been observed in the progression from chronic pancreatitis to PDAC [3]. Additionally, the presence of intraductal papillary mucinous neoplasms (IPMN), mucinous cystic neoplasms (MCN), or pancreatic intraepithelial neoplasia (PanIN) confers an increased risk as well. The risk associated with these lesions varies based on factors such as size, location, histology, and biochemistry, necessitating either surveillance or surgical resection [4].

In recent years, the role of the microbiome in tumor development has gained more interest [5–8]. Although the human microbiota contributes to disease prevention by enhancing nutritional and hormonal balance, modulating inflammatory responses, detoxifying harmful substances, and producing beneficial metabolites [9], recent studies reveal its crucial roles in malignancies such as leukemia [10], colorectal [11, 12], liver [13], lung [14], and pancreatic cancer [15]. Elevation of bacterial 16S rRNA can be found in precursor lesions [16, 17] such as IPMNs, and although studies have attempted to identify a specific microbiome signature for PDAC patients, no universally distinctive pattern has been consistently observed or agreed upon, with findings often varying due to differences in study populations, methodologies, and analytical approaches [18, 19]. Notably, patients with tumors located in the pancreatic head and concurrent biliary obstruction exhibited a more distinct microbiome, emphasizing the influence of the biliary microbiome on the carcinogenesis of pancreatic cancer [20, 21]. Along the oro-intestinal axis, the oral microbiota could potentially impact pancreatic carcinogenesis and may serve as a prognostic or diagnostic biomarker for pancreatic cancer [7, 22].

This systematic review will summarize the microbiome profiles of stool, saliva, bile and tissue in patients with CP, precursor lesions, pancreatic cancer, and healthy controls. Key findings will include bacterial taxa that are altered in these groups, and abundances of specific taxa will be highlighted, along with potential shifts in bacterial populations that may indicate a progression from chronic pancreatitis or precursor lesions to cancer. The primary objective is to identify bacterial taxa involved in this process, including those that might inhibit carcinogenesis. We are confident that the results of this literature review will contribute to understanding the role of the microbiome in pancreatic cancer and proposing potential new biomarkers for early cancer detection.

## Methods

This systematic review will be conducted according to the recommendation of the Preferred Reporting Items for Systematic reviews and Meta-Analyses (PRISMA) guidelines (for the PRISMA-P checklist, see additional file 1) [23], the recommendations from the Study Center of the German Society of Surgery [24], and as outlined in a predefined protocol (PROSPERO: CRD42023487995). The eligibility criteria for this systematic review are defined using the PICO-framework:

- Population
  - ◦ Adult human patients diagnosed with chronic pancreatitis, pancreatic cancer, or precursor lesions (e.g., IPMN, MCN)
  - ◦ Studies must provide baseline diagnostic criteria or staging methods
- Intervention
  - ◦ Studies must provide microbiome profiling of at least one of the following sample types: saliva, stool, bile, pancreatic fluid, and pancreatic or tumoral tissue.
  - ◦ Accepted microbiome methods include 16 s rRNA sequencing. Whole genome shotgun metagenomics, qPCR, MALDI-TOF, or other culture-based techniques.
  - ◦ Studies that allow identification of bacterial taxa to at least the genus level will be prioritized. However, studies with lower taxonomic resolution (e.g., phylum or class) will be considered if they report relevant diversity metrics (e.g., alpha or beta diversity) or disease-specific comparisons.
- Comparison
  - ◦Studies that compare microbiome data from patients with chronic pancreatitis, pancreatic precursor lesions (IPMN, MCN, PanIN), or pancreatic cancer (PDAC) to matched or unmatched healthy controls without pancreatic disease will be included. Comparisons must involve human samples and demonstrate either compositional or diversity-based differences between diseased and healthy cohorts. Studies comparing microbiome profiles solely between disease groups (e.g., CP vs. PDAC) without inclusion of a healthy control group will be excluded.
- Outcome
  - ◦ Alpha diversity (e.g., Shannon index, Chao1, Simpson index)
  - ◦ Beta diversity (e.g., Bray–Curtis, UniFrac distance)
  - ◦ Differential abundance of bacterial taxa between groups
  - ◦ The presence or absence of specific bacterial entities

All stages of study selection, data extraction, and quality assessment will be carried out independently by two reviewers: M. P. and A. W. Any disagreements will be resolved by consulting a third reviewer (F. P.) following the recommendations of the Cochrane Collaboration [25].

### Systematic literature search

A systematic literature search will be performed using validated methods of the Cochrane Collaboration and the Study Center of the German Society of Surgery [25]. The search will not be restricted to specific languages or years of publication. The following databases will be searched: the Cochrane Central Register of Controlled Trials (CENTRAL), MEDLINE (via PubMed), Embase, and Web of Science. The search strategy was designed by an experienced information specialist (E. K.) and is based on combinations of Medical Subject Heading (MeSH) terms and text words for each database, as shown below


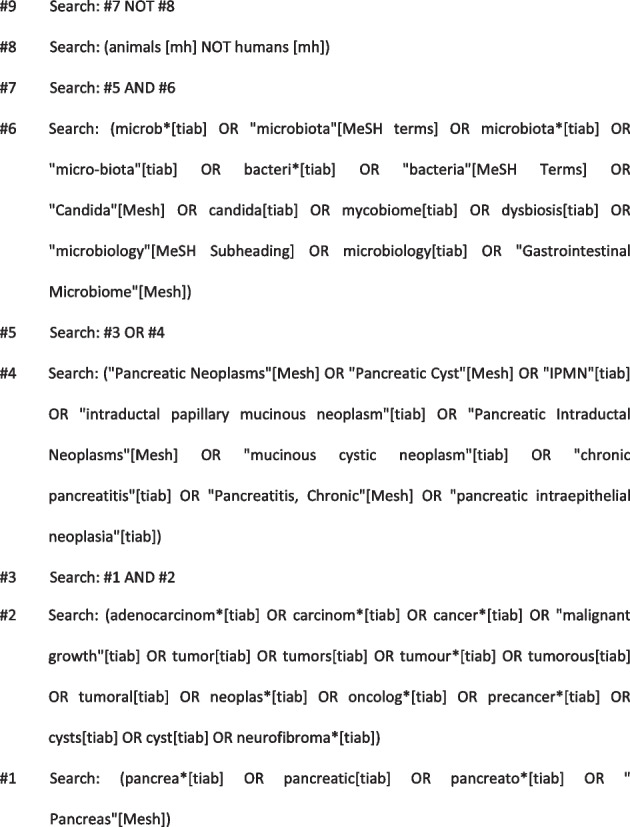


In addition to database searches, we will screen the reference lists of all included articles to identify relevant studies not captured through database queries. We will also consider grey literature sources, including Google Scholar and relevant conference proceedings, when available. If potentially relevant unpublished or preprint data are identified, these will be evaluated for eligibility.

### Study selection

Studies meeting the following inclusion criteria will be included in the review after abstract and full-text screening: Randomized controlled trials (RCT), prospective comparative cohort studies, retrospective comparative cohort studies, studies investigating microbial contents in saliva, bile, pancreatic fluid, pancreatic tissue and stool among patients diagnosed with chronic pancreatitis, precancerous pancreatic lesions (IPMN, MCN, PanIN), and pancreatic cancer.

Studies will be compared based on their methodologies, including sample collection, bacterial identification techniques (e.g., MALDI-TOF, sequencing methods such as shotgun-seq or 16 s, qPCR), and data analysis approaches (bioinformatic pipeline). Quantitative and qualitative assessment of bacterial populations and comparative analyses between different patient groups with correlation to clinical outcome and survival will be prioritized as well as studies that differentiate bacterial taxonomy to at least the class level (class, family, genus, species, and, if possible, subspecies).

Conversely, trials that do not focus specifically on bacterial content or do not provide a clear comparison between patient samples will be excluded. Studies comparing microbiome profiles across non-pancreatic cancer or non-chronic pancreatitis populations or those examining the impact of external factors (e.g., diet, probiotics) without direct relevance to chronic pancreatitis, pancreatic precursor lesions, and pancreatic cancer will be omitted. Research investigating nonbacterial microbiota (e.g., parasites, viruses, fungi) without concurrent bacterial analysis or focusing on single bacterial strains will also be excluded. In-vitro cell culture, animal studies, meeting abstracts, letters/comments/editorials, and publications for which the full text is irretrievable will be excluded.

Prior to formal screening, a calibration exercise will be conducted in which all reviewers independently assess a subset of records. This process will help refine the eligibility criteria, ensure a shared understanding of inclusion and exclusion parameters, and improve inter-reviewer consistency.

### Data extraction and management

Data will be extracted from the trials that meet our final inclusion criteria using a standardized form in Microsoft Excel (Microsoft Corporation, Redmond, WA, USA). The form will be developed in alignment with the review objectives and refined after piloting on the first three eligible studies. Study screening through title, abstract, and full-text phases will be performed in Covidence (Veritas Health Innovation, Melbourne, Australia). Two authors will independently extract data to ensure quality and consistency. Any discrepancies will be resolved by a third reviewer. A final extraction sheet will be determined for database entry. The following trial characteristics will be extracted:

1. Methods: Author, year and journal of publication, country of origin, trial design, total duration of the trial, sample size calculation, analysis methods, bioinformatic pipeline
2. Participants: Age, gender, body mass index, diagnosis (underlying disease), inclusion criteria, and exclusion criteria
3. Samples: Saliva, bile, pancreatic fluid, stool, PDAC tissue, and healthy tissue
4. Microbiome methods: Sequencing or detection technique (e.g., 16S rRNA, WGS, qPCR, MALDI-TOF), taxonomic depth reported, and reference database used for classification
5. Outcomes: Alpha-diversity metrics (e.g., Shannon, Simpson, Chao1), beta-diversity or compositional differences (e.g., UniFrac, Bray–Curtis), differential abundance of taxa (e.g., enriched or depleted genera/species), and the presence or absence of specific bacterial taxa
6. Notes: Funding for trial and notable conflicts of interest of trial authors

### Endpoints of the systematic review

The main aim of this study is to describe bacterial diversity, including alpha and beta diversity, among patients with chronic pancreatitis, pancreatic precursor lesions, and pancreatic cancer to understand differences in the microbial composition associated with these conditions in comparison to healthy controls. The study intends to identify specific bacterial taxa that exhibit differential abundance or depletion across these different disease states. The extent of diagnosis-related dysbiosis will also be evaluated, if possible. Finally, the association between specific bacterial profiles and clinical outcomes will be assessed to understand the potential impact of the microbiome on critical endpoints, such as overall survival or disease stage.

**Fig. 1:**
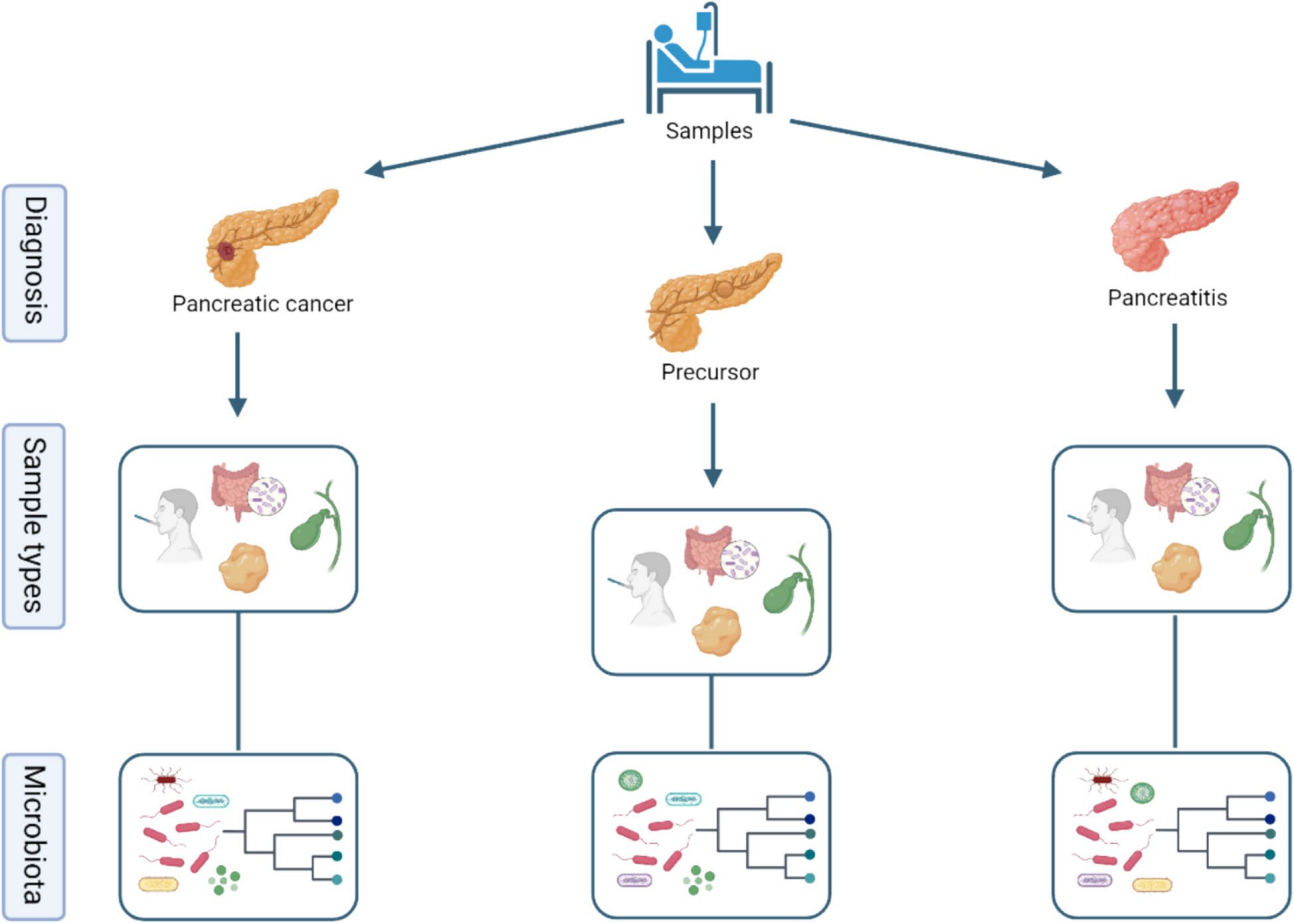
Overview — collection of samples and microbiota analysis (created in https://BioRender.com)

These endpoints provide a comprehensive framework for analyzing the relationship between microbiome and pancreatic cancer, considering various aspects such as bacterial diversity, specific taxa, dysbiosis, and clinical outcomes (Fig. 1).

### Qualitative assessment and critical appraisal of included trials

The methodological quality of included studies will be assessed independently by two review authors using several tools for assessing the risk of bias depending on included studies: ROBINS-I tool [26], CHARMS [27], PROBAST [28], or Cochrane Risk of Bias 2.0 tool (RoB2) [29].

For randomized controlled trials (RCTs), we will apply the Cochrane Risk of Bias 2.0 tool (RoB 2), evaluating domains such as randomization process, deviations from intended interventions, missing outcome data, measurement of the outcome, and selection of the reported result.

For non-randomized interventional studies, including observational cohort and case–control studies, we will use the ROBINS-I tool (Risk Of Bias in Non-randomized Studies of Interventions), which evaluates bias due to confounding, selection, classification of interventions, deviations from intended interventions, missing data, measurement of outcomes, and selection of reported results.

For prediction model studies, if applicable, the CHARMS checklist and PROBAST tool will be used.

All assessments will be performed at the study level and, where applicable, at the outcome level. The results of the risk-of-bias assessment will be incorporated into the interpretation of findings and will inform the GRADE [30] evaluation of the certainty of evidence for each outcome. The certainty of evidence will be assessed using the GRADE approach (Grading of Recommendations, Assessment, Development and Evaluation) for each predefined outcome.

The following five domains will be evaluated:

- Risk of bias
- Inconsistency (heterogeneity of results across studies)
- Indirectness (relevance of study population, microbiome methods, or outcomes to the review question)
- Imprecision (width of confidence intervals and sample size)
- Publication bias (assessed via funnel plots if ≥ 10 studies)

Each outcome will be rated as having high, moderate, low, or very low certainty of evidence. In cases where meta-analysis is feasible, GRADE will be applied to pooled results.

Summary of Findings (SoF) tables will be created using GRADEpro software to present the overall certainty of evidence alongside effect estimates for each outcome.

### Data synthesis and statistical analysis

Data and outcomes from the included studies will first be thoroughly explored using descriptive statistics. Pooling of individual study effects through meta-analysis methods will only be performed if the identified studies are similar enough concerning, e.g., disease, bacteria detection method, and patient cohort. Any data synthesis or pooling will be performed within groups based in disease status, bacterial classification, and origin sample.

For the meta-analysis of single groups, the single proportions per trial will be used as the effect measure and will be reported along the respective 95% confidence intervals. Continuous outcomes will be extracted and pooled with their means and standard deviations, using the inverse variance method. A random intercept logistic regression model will be utilized to estimate the overall proportion and to account for expected between-trial heterogeneity. Group comparisons (e.g., PDAC vs. healthy controls) will be analyzed using odds ratios (ORs) or standardized mean differences, depending on the outcome format.

Statistical heterogeneity among the effect estimates of the included trials will be evaluated using the between-study variance *τ*^2^ and the *I*^2^ statistic. Furthermore, prediction intervals will be calculated to investigate present heterogeneity. If substantial heterogeneity is detected (e.g., *I*^2^ > 75%), pooling will be avoided, and subgroup or sensitivity analyses will be performed to explore sources of heterogeneity.

Studies with different methodological designs (e.g., observational vs. interventional studies) will not be pooled together in meta-analyses but will be analyzed in design-specific subgroups. If quantitative synthesis is not feasible for specific outcomes due to heterogeneity in reporting or methodology, we will conduct a structured narrative synthesis, grouped by disease entity, sample type, and microbiome analysis technique.

Primary statistical analysis and meta-analysis will be performed using R (version 4.2.1 or higher). Forest plots will be used for graphical presentation of (overall) effect estimates, while publication bias will be explored using funnel plots if 10 or more studies are included in an analysis.

## Discussion

This systematic review aims to investigate the complex relationship between the microbiome and the development and progression of pancreatic cancer. By examining the bacterial diversity among patients with chronic pancreatitis, pancreatic precursor lesions, and pancreatic cancer, we seek to identify specific microbiome signatures associated with each disease state.

The correlation between specific bacterial taxa with disease progression is particularly important as it may provide insights into the mechanisms through which the microbiome influences carcinogenesis. For instance, a shift in the microbial composition from chronic pancreatitis to precursor lesions and subsequently pancreatic cancer could indicate a progressive dysbiosis that promotes malignancy. Such microbial transitions have been described in other diseases, e.g., inflammatory bowel disease or colon cancer [12, 31–34], but have not yet been thoroughly investigated in pancreatic cancer. This underscores the importance of the proposed review, as we aim to explore and describe these potential microbial changes within the context of pancreatic cancer. Understanding these dynamics could lead to preventive strategies or interventions that modulate the microbiome to halt or reverse disease progression.

Quantifying bacterial load and evaluating dysbiosis will deepen the understanding of microbial imbalances in pancreatic diseases. High bacterial load or pronounced dysbiosis may correlate with more aggressive disease phenotypes, providing additional markers for prognosis. Moreover, identifying bacterial species that are protective against pancreatic cancer could lead to new avenues for probiotic or microbiome-based therapies.

The association of specific bacterial profiles with clinical outcomes such as disease stage, progression-free survival, and overall survival is crucial for translating microbiome research into clinical practice. If certain bacterial taxa are identified to predict clinical outcomes, they could be incorporated into risk stratification models to guide treatment decisions.

However, several limitations must be considered. The variability in microbiome sampling methods, sequencing techniques, data reporting, and data analysis across studies may introduce heterogeneity that complicates comparisons. Additionally, the cross-sectional nature of most studies limits the ability to infer causality. Longitudinal studies and randomized controlled trials are needed to establish causal relationships and the impact of microbiome modulation on clinical outcomes. Regarding methodology, the risk of bias due to blinding measures and funding sources will be carefully evaluated. Blinding is often challenging in microbiome research due to the nature of sample collection and analysis. Therefore, the feasibility of blinding and potential biases will be assessed according to established guidelines.

In conclusion, this systematic review will provide comprehensive insights into the role of the microbiome in pancreatic cancer. By identifying key bacterial taxa and understanding their association with disease progression and clinical outcomes, we are determined to contribute to the development of microbiome-based diagnostics and therapeutics in the management of pancreatic cancer.

## Supplementary Information

Additional file 1. PRISMA-P 2015 Checklist.

## Data Availability

The datasets used and/or analyzed during the current study are available from the corresponding author on reasonable request.

## Abbreviations

CP: Chronic pancreatitis
CENTRAL: The Cochrane Central Register of Controlled Trials
CHARMS: Checklist for critical Appraisal and data extraction for systematic Reviews of prediction Modelling Studies
GRADE: Grades of Recommendation, Assessment, Development, and Evaluation
IPMN: Intraductal papillary mucinous neoplasm
MCN: Mucinous cystic neoplasm
MeSH: Medical Subject Headings
PaniN: Pancreatic intraepithelial neoplasia
PDAC: Pancreatic ductal adenocarcinoma
PRISMA: Preferred Reporting Items for Systematic reviews and Meta-Analyses
PROBAST: Prediction model Risk-Of-Bias Assessment Tool
PROSPERO: International Prospective Register of Systematic Reviews
RCT: Randomized controlled trial
rRNA: Ribosomal DNA
RoB2: Cochrane risk-of-bias tool for randomized trials
ROBINS-I: Risk Of Bias In Non-randomized Studies Of Interventions
